# Maternal overweight but not paternal overweight before pregnancy is associated with shorter newborn telomere length: evidence from Guangxi Zhuang birth cohort in China

**DOI:** 10.1186/s12884-021-03757-x

**Published:** 2021-04-09

**Authors:** Bincai Wei, Yantao Shao, Jun Liang, Peng Tang, Meile Mo, Bihu Liu, Huishen Huang, Hui Juan Jennifer Tan, Dongping Huang, Shun Liu, Xiaoqiang Qiu

**Affiliations:** 1 Department of Epidemiology and Health Statistics, School of Public Health, Guangxi Medical University, No. 22 Shuangyong Road, Nanning, 530021 Guangxi China; 2 Department of Student Work, The First Clinical Medical College of Guangxi Medical University, Nanning, 530021 Guangxi China; 3 Yong Loo Lin School of Medicine, National University of Singapore, 10 Medical Dr, Singapore, 117597 Singapore; 4 Department of Sanitary Chemistry, School of Public Health, Guangxi Medical University, No. 22 Shuangyong Road, Nanning, 530021 Guangxi China; 5 Department of Maternal, Child and Adolescent Health, School of Public Health, Guangxi Medical University, No. 22 Shuangyong Road, Nanning, 530021 Guangxi China

**Keywords:** Parents, Pre-pregnancy body mass index, Telomere length, Cord blood, Newborns

## Abstract

**Background:**

Telomere length (TL) is variable at birth and is inversely associated with body mass index (BMI) in adulthood. A growing number of evidences suggested that a higher maternal pre-pregnancy BMI results in adverse offspring health outcomes, especially shorter newborn TL. However, a newborn’s genetic endowment is equally derived from both parents, the association between parental pre-pregnancy BMI and newborn TL has been rarely discussed. We aimed to determine the association between parental pre-pregnancy BMI and newborn TL.

**Methods:**

A total of 1082 parent-newborn pairs were recruited from the Guangxi Zhuang Birth Cohort (GZBC). TL in cord blood was measured using quantitative real-time polymerase chain reaction (qPCR) and expressed as the ratio of telomere copy number to single-copy gene number (T/S). A series of linear regressions were performed to assess the associations between parental pre-pregnancy BMI and newborn TL.

**Results:**

Mothers who were overweight before pregnancy had significantly shorter cord blood telomere length in their newborns than those who were normal weight before pregnancy [percentage change: − 7.96% (95% CI: − 14.49 to − 0.69%; *P* = 0.032)]. Further analysis of the combined effects of parental weight status on newborn TL showed that TL was significantly shortened among newborns whose mothers were overweight and fathers were of healthy weight when compared with those whose mothers and fathers were both of normal weight [percentage change: − 8.38% (95% CI: − 15.47 to − 0.92%; *P* = 0.028)]. Subgroup analysis indicated these effects were more pronounced among male newborns and those whose paternal age < 31 years or maternal age ≥ 28 years at delivery.

**Conclusions:**

Maternal pre-pregnancy overweight, but not paternal pre-pregnancy overweight is associated with shorter newborn TL. Weight control in reproductive women and effective healthy weight management before pregnancy may be of particular benefit for improving longevity and life quality of offspring.

**Supplementary Information:**

The online version contains supplementary material available at 10.1186/s12884-021-03757-x.

## Background

The phenomenon of overweight and obesity has been a heated topic in the past decades, which is a major public health problem of most countries in the world, including China. With the concept of “Giving better birth and care” spreading in China, overweight and obesity before pregnancy has gradually attracted more attention. Previous studies had reported that higher parental pre-pregnancy BMIs were associated with adverse offspring outcomes, such as higher risk of macrosomia [1], adverse body composition after birth [2], type 1 diabetes [3], higher blood pressure [4] and so on.

The telomere is a terminal section of a chromosome, which contains a number of repeats of the sequence TTAGGG, and it is responsible for maintaining chromosome integrity and genome stability [5]. Naturally, the telomeres are gradually shortened when a cell undergoes DNA replication and cell division. For this reason, scientists believe that telomere length (TL) is not only a biomarker of biological aging, but also a “telomeric clock” [6] and a prediction of life span [7, 8]. TL has been shown to be associated with several age-related diseases, such as type 2 diabetes [9], cardiovascular disease [10] and cancer [11]. Considering the importance of telomeres, clarifying the related factors of TL is of great significance for maintaining the normal function of telomeres.

A previous study showed that TL is hereditary and largely determined before adulthood, and early life environmental factors are the main determinants of TL throughout the course of human life [12]. The early life determinants are mainly from maternal factors, such as maternal stress [13], maternal menarche age [14], maternal second-hand smoke exposure [15], and environment risk such as cadmium exposure [16]. In relation to pre-pregnancy overweight and obesity, the effect of maternal BMI on the TL of the offspring has been well characterized [17]. However, the genetic endowment of a newborn is equally derived from both parents. It remains unclear whether paternal pre-pregnancy overweight and obesity has an effect on newborn TL, and whether parental pre-pregnancy weight status has interactive effects on newborn TL. In the present study, we aimed to investigate the association of parental pre-pregnancy BMI with newborn TL.

## Methods

### Study population and data collection

Guangxi is the ninth largest province with over 49.2 million residents in southwestern China, however, the per capita Gross regional product (GRP) of Guangxi was only ranked 26th to 28th among China’s 31 provinces from 2003 to 2017 [18]. Data between June 2015 and May 2018 for this study were extracted from the Guangxi Zhuang Birth Cohort (GZBC), which was conducted in 6 county-level hospitals with visitors mainly from rural areas, and details on the ongoing GZBC have been introduced in our previous study [19]. In the present study, a total of 1239 mothers were extracted out for TL detection. However, 157 participants were excluded due to twins (*n* = 15) and stillbirth (*n* = 9), missing information on parental BMI (*n* = 38), unavailable cord blood DNA sample for TL measurement (*n* = 69), and large variabilities TL measurements between duplicate measurements for cord blood telomere (*n* = 26) (Flowchart was presented in Additional file 1). A total of 1082 parent-newborn pairs (singletons) with full data and cord blood telomere were included for the final analyses. This study protocol was approved by the ethical committee of Guangxi Medical University (No.20140305–001). Written informed consent was obtained from all participants.

On the first antenatal visit, the personal information was collected at the hospitals by professionally trained interviewers through a standardized and structured questionnaire. Information on maternal factors around pregnancy and newborns was obtained from medical data. Gestational age (weeks) was estimated based on ultrasound data or the date of last menstrual period self-reported by the pregnant women. Pre-pregnancy weight and height measured on the first antenatal visit was self-reported by mothers and fathers, respectively. BMI was calculated as weight /height^2^ (kg/m^2^). The categories of parental BMIs were based on the BMI cut-offs for Chinese adults recommended by the Working Group of Obesity in China [20]. BMI < 18.5 kg/m^2^, 18.5 ≤ BMI <24.0 kg/m^2^, 24.0 ≥ BMI < 28.0 kg/m^2^, and BMI ≥28.0 kg/m^2^, was defined as underweight, normal weight, overweight and obesity, respectively.

### Cord blood collection and relative telomere length measurement

Cord blood was drawn into 5 mL EDTA tubes immediately after delivery. Samples were centrifuged at 4000 rpm for 10 min and blood tubes were stored at − 80 °C. Genomic DNA extraction kit (Aidlab Biotechnologies) was used to extract DNA from leukocytes of umbilical cord blood according to the manufacturer’s instructions. DNA samples with the A260/A280 ratio within the range 1.8 to 2.0 were considered eligible. A real-time quantitative polymerase chain reaction (qPCR) method described by Cawthon [21] was used to examined the relative TL in each DNA sample. Relative TL was determined as the ratio of telomere repeat copy number (T) to a single copy gene (36B4) copy number (S). The primers sequences of telomeric gene and single-copy gene (36B4) were as follows. Forward primer of telomeric gene: 5′-CGGTTTGTTTGGGTTTGGGTTTGGGTTTGGGTTTGGGTT-3′; reverse primer of telomeric gene: 5′-GGCTTGCCTTACCCTTACCCTTACCCTTACCCTTACCCT-3′; forward primer of 36B4: 5′-CAGCAAGTGGGAAGGTGTAATCC-3′; reverse primer of 36B4: 5′-CCCATTCTATCATCAACGGGTACAA-3′. For both telomeric gene and single copy gene (36B4), reaction mixture (20 μL) contained 1 μL of genomic DNA (10 ng/μL), 10 μL of PowerUp SYBR Green Master Mix (2X), 100 nM of the forward, and 100 nM of the reverse; RNase free water was added to the final volume. For the telomere PCR reactions, the cycling conditions were 2 min at 50 °C, 2 min at 95 °C, followed by 40 cycles of 95 °C for 15 s, then 62 °C for 1 min. For the 36B4 PCR reactions, the cycling conditions were 2 min at 50 °C, 2 min at 95 °C, followed by 40 cycles of 95 °C for 15 s, 62 °C for 20 s, and 72 °C for 1 min. Each sample was measured in duplicates for both T and S, and was performed in separate 96-well plates using a StepOnePlus real-time PCR system (Applied Biosystems). A standard reference genomic DNA sample was randomly selected from all quality samples. The standard curve, plotted using the standard reference genomic DNA sample, was utilized by two-fold serially dilution of one reference DNA sample to generate a 7-point curve with DNA concentration ranging from 3.125 to 200 ng/μL (R^2^ ≥ 0.98). Replicates of each plate were done to ensure reliable values. The qPCR amplification efficiency values were 98% for T and 101% for S. The coefficient variations within the duplicates were 0.47% for the telomere runs and 0.61% for the single copy runs. A positive standard reference genomic DNA sample and a negative standard reference genomic DNA sample in each 96-well plate was set to calculate the △Ct for each tested sample. The 2^-△△Ct^ method was used to calculate the average relative TL [22].

### Statistical analysis

Mean (SD) was used to describe the normally distributed continuous variables, median (interquartile range, IQR) for skewed data, and the numbers (percentages) was used to describe the categorical variables. The relative cord blood TL showed skewed distribution and were Log10 transformed to improve normal distribution. To study potential confounding structure in our dataset, we used ANOVA and Chi-square test to assess the distributions of continuous variables and categorical variables across the 3 categories of maternal pre-pregnancy BMI (underweight, normal weight, and overweight), respectively. Due to the small number of underweight fathers (1.75%), we combined underweight and normal weight parents to make up the group of healthy weight (BMI < 24.0 kg/m^2^) parents in the analysis of the impact of paternal BMI and the combined effects of parental weight status on newborn TL.

Pearson correlation and restricted cubic spline analysis was used to detect the linear and non-linear relationship between parental BMIs and newborns TL, respectively. Multiple linear regression models were also conducted to examine the association between parental BMI categories and newborn TL. We selected covariates based on prior knowledge, which included parental demographic characteristics (parental age at delivery, weight and height), and maternal lifestyles (drinking and smoking before pregnancy, passive smoking during pregnancy). Maternal factors around pregnancy, included residential place, parity, gravidity, pregnancy comorbidities or complications (diabetes, hypertension, pre-eclampsia, hyper- or hypothyroidism, digestive system diseases and blood system diseases), and cesarean section. Newborns’ factors, included sex, gestational age, and anthropometric measures. In model A, we adjusted for parental age at delivery and newborns’ factors. In model B, we additionally adjusted for maternal factors around pregnancy. Since only two mothers smoked before pregnancy, the factor of smoking before pregnancy was not included in the adjusted models. As previous studies showed effects of paternal age and newborn sex on newborn TL [6, 17], we also performed subgroup analysis stratified by newborn sex, the median age of mothers (28 years) and fathers (31 years). To evaluate the robustness of our study, we performed sensitivity analyses that excluded mothers who drank before pregnancy, those who underwent passive smoking during pregnancy, those who experienced pregnancy comorbidities or complications, and newborns with preterm birth or low birth weight. Considering that air pollution has an important impact on TL [23, 24], we also excluded participants from urban areas. The relationship between parental BMI and newborn TL was expressed as a regression coefficient (*β* value) and corresponding 95% confidence interval (CI). To express the relative change in newborn TL in each model, we calculated the percentage change as follow: percentage change = (10^**β**^-1) × 100%. The SPSS software version 25.0 and R version 4.0.2 were used to carry out all statistical tests, and *P*-value< 0.05 was considered as statistically significant.

## Results

### Study participants

All in all, the mean ages of mothers and fathers at delivery were 28.5 years and 31.4 years, respectively. The mean pre-pregnancy BMIs of mothers and fathers were 20.2 (2.3) kg/m^2^ and 22.1 (1.4) kg/m^2^, respectively. The number of overweight participants was 74 (6.8%) and 87 (8.0%) for mothers and fathers, respectively. Additionally, there were no obese parents and only 2 mothers smoked before pregnancy, and 1013 (93.6%) mothers from rural areas. The mean birth weight of newborns was 3120.3 (384.0) g. Among 1082 newborns, 582 were male, accounting for 53.8%. The gestational age of the newborns was from 34 to 42 weeks, and the average gestational age was 38.66 (1.24) weeks, and 40 of them were preterm birth (gestational age < 37 weeks). Higher maternal pre-pregnancy BMI was significantly associated with increased newborn birth weight, maternal age and paternal age (all *P* < 0.001). The median (IQR) of newborns’ relative cord blood TL was 1.058(0.877,1.265). The baseline characteristics of participants according to maternal pre-pregnancy BMI categories are showed in Table 1.

**Table 1:** Characteristics of parent-newborn pairs according to maternal pre-pregnancy BMI

Characteristics	UW (*n* = 255)	NW (*n* = 753)	OW (*n* = 74)	*P*-value
**Newborn**				
sex				0.008
Female	139 (54.5%)	331 (44.0%)	30 (40.5%)	
Male	116 (45.5%)	422 (56.0%)	44 (59.5%)	
Gestational age, weeks	38.67 (1.19)	38.65 (1.26)	38.68 (1.23)	0.963
Birth weight, g	3006.75 (360.39)	3144.57 (379.27)	3264.05 (421.81)	< 0.001
**Maternal**				
Age, years	26.68 (4.99)	28.75 (5.12)	32.46 (4.50)	< 0.001
Residential place				0.658
Urban areas	14 (5.5)	49 (6.5)	6 (8.1)	
Rural areas	241 (94.5)	704 (93.5)	68 (91.9)	
Parity, n				< 0.001
1	138 (54.1%)	283 (37.6%)	19 (25.7%)	
≥ 2	117 (45.9%)	470 (62.4%)	55 (74.3%)	
Gravidity, n				< 0.001
1	82 (32.2%)	145 (19.3%)	8 (10.8%)	
≥ 2	173 (67.8%)	608 (80.7%)	66 (89.2%)	
Drinking pre-pregnancy, n				0.361
No	248 (97.3%)	733 (97.3%)	74 (100%)	
Yes	7 (2.7%)	20 (2.7%)	0 (0.0%)	
Smoking pre-pregnancy, n				0.657
No	254 (99.6%)	752 (99.9%)	74 (100%)	
Yes	1 (0.4%)	1 (0.1%)	0 (0.0%)	
Passive smoking during pregnancy, n				0.919
No	97 (38.0%)	294 (39.0%)	30 (40.5%)	
Yes	158 (62.0%)	459 (61.0%)	44 (59.5%)	
Pregnancy comorbidities or complications, n				0.081
No	231 (90.6%)	659 (87.5%)	60 (81.1%)	
Yes	24 (9.4%)	65 (12.5%)	14 (18.9%)	
Cesarean section, n	59 (23.1%)	220 (29.2%)	24 (32.4%)	0.119
**Paternal**				
Age, years	29.33 (5.20)	31.72 (5.60)	35.49 (4.96)	< 0.001
Drinking pre-pregnancy, n				0.204
No	46 (18.0)	99 (13.1)	14 (18.9)	
Yes	117 (45.9)	338 (44.9)	31 (41.9)	
Missing	92 (36.1)	316 (42.0)	29 (39.2)	
Smoking pre-pregnancy, n				0.366
No	63 (24.7)	192 (25.5)	18 (24.3)	
Yes	100 (39.2)	245 (32.5)	27 (36.5)	
Missing	92 (36.1)	316 (42.0)	29 (39.2)	
BMI, kg/m^2^	21.85 (1.61)	22.17 (1.36)	22.24 (1.62)	0.007
BMI categories, n				0.751
HW	237 (92.9%)	691 (91.8%)	67 (90.5%)	
OW	18 (7.1%)	62 (8.2%)	7 (9.5%)	

Values are presented as mean (SD), n (%)

Abbreviation: *BMI* Body mass index, *UW* Underweight, *NW* Normal weight, *OW* Overweight, *HW* Healthy weight (normal and underweight)

### Association between parental pre-pregnancy BMI and newborn TL

As there was no significant relationship found between parental BMI and newborn TL after conducting the Pearson correlation and restricted cubic spline analysis (Additional files 2 and 3), we used the multivariate linear regression model to test the associations by transforming parental BMI into categorical variables. As shown in Table 2, the newborn TL of overweight mothers were significantly shortened by 8.17% (95%CI, − 14.89 to − 0.92%; *P* = 0.026) after adjusting for parental age at delivery and newborn factors (newborn sex, gestational age and birth weight) when compared to normal weight mothers. Even after additionally adjusting for maternal factors (residential place, gravidity, parity, drinking before pregnancy, passive smoking during pregnancy, pregnancy comorbidities or complications, and cesarean section), a significant difference [percentage change: -7.96% (95%CI, − 14.49 to − 0.69%; *P* = 0.032)] was still observed. However, there was no significant effect found of paternal BMI on newborn relative TL in cord blood. Further analysis of the combined effect of parental BMI on newborn TL showed that TL was significantly shortened by 8.38(95%CI, − 15.47 to − 0.92%; *P* = 0.028) among the newborns with overweight mothers and fathers of healthy weight when compared with those whose mothers and fathers were both of normal weight in the fully adjusted model. In addition, we had considered paternal lifestyle factors into the analysis between parental pre-pregnancy BMI and newborn TL, however, there was a lot of missing data (*n* = 437) about paternal lifestyle factors (drinking and smoking before pregnancy) and there was still a trend that maternal pre-pregnancy overweight was inversely associated with newborn TL, although no statistical significance (Additional file 4).

**Table 2:** Categorized analysis between parental pre-pregnancy BMI and newborn TL

All (*n* = 1082)	Unadjusted		Model A		Model B	
	Percentage change (95% CI)	*P*-value	Percentage change (95% CI)	*P*-value	Percentage change (95% CI)	*P*-value
Maternal BMI						
NW	Ref		Ref		Ref	
UW	−0.23(−4.50,4.23)	0.937	0.23(−4.28,4.71)	0.958	0.23(−4.28,4.95)	0.929
OW	−7.96(−14.49, − 0.92)	0.029	−8.17(− 14.89, − 0.92)	0.026	− 7.96(− 14.49, − 0.69)	0.032
Paternal BMI						
HW	Ref		Ref		Ref	
OW	0.69(−6.03,7.65)	0.850	0.93(−5.59,8.14)	0.785	0.93(−5.59,8.14)	0.772
Parents’ weight status combination						
Both parents HW	Ref		Ref		Ref	
OW father, HW mother	0.46(−6.24,7.89)	0.892	0.69(−6.24,7.89)	0.869	0.69(−6.24,8.14)	0.866
OW mother, HW father	−8.17(−14.89, −0.92)	0.030	− 8.59(− 15.67, − 1.14)	0.024	−8.38(− 15.47, − 0.92)	0.028
Both parents OW	−4.50(−23.97,20.23)	0.698	−3.39(− 23.26,21.62)	0.768	−2.50(−22.55,22.74)	0.830

Abbreviation: *BMI* Body mass index, *TL* Telomere length, *UW* Underweight, *NW* Normal weight, *HW* Healthy weight (normal and underweight), *OW* Overweight

Model A: adjusted for parental age, newborn factors (sex, gestational age and birth weight)

Model B: Model A + maternal factors (residential place, gravidity, parity, drinking before pregnancy, passive smoking during pregnancy, pregnancy comorbidities or complications, and cesarean section)

Estimates are presented as a percentage change in average relative telomere length

### Stratified analysis by newborn sex and parental age

We further conducted stratified analysis by newborn sex and parental age. We found that the association between overweight mothers and newborn TL was more pronounced among male newborns (Table 3). Even after additional adjusting for maternal factors, a trend still existed between overweight mothers and shortened newborn TL [percentage change: -9.01% (95%CI: − 17.40 to 0.23%; *P* = 0.054)]. We found a trend that the combined effect of parental weight status on newborn TL tend to be more obvious among male newborns in the fully adjusted model [percentage change: -8.80% (95%CI: − 17.59 to 0.69%; *P* = 0.068)]. However, we found no significant effect of paternal BMI on newborn TL in cord blood. When stratifying by parental age, we found single effects of maternal BMI and the combined effect of parental BMI on newborn TL were more pronounced among those whose paternal age < 31 years or those whose maternal age ≥ 28 years (Tables 4 and 5, respectively). However, no significant interactions were found between parental pre-pregnancy BMI and newborn sex or parental age (all *P* for interaction > 0.10) on the changes of newborn TL (Additional file 5).

**Table 3:** Associations between parental pre-pregnancy BMI and newborn TL, stratified by newborn sex

Subgroup	Unadjusted		Model A		Model B	
	Percentage change (95% CI)	*P*-value	Percentage change (95% CI)	*P*-value	Percentage change (95% CI)	*P*-value
**Male (*n*** **= 582)**						
Maternal BMI						
NW	Ref		Ref		Ref	
UW	0.69(− 5.38,7.15)	0.839	0.23(− 6.03,6.91)	0.934	0.46(− 5.81,7.15)	0.905
OW	−9.84(− 17.96, − 0.92)	0.033	−9.22(− 17.59,0.00)	0.048	−9.01(− 17.40,0.23)	0.054
Paternal BMI						
HW	Ref		Ref		Ref	
OW	2.33(−7.32,12.72)	0.655	2.57(− 7.10,12.98)	0.616	2.09(− 7.53,12.72)	0.686
Parents’ weight status combination						
Both parents HW	Ref		Ref		Ref	
OW father, HW mother	2.80(−7.10,13.76)	0.588	3.04(−6.89,14.02)	0.568	2.33(−7.74,13.24)	0.670
OW mother, HW father	−9.43(−17.78, −0.23)	0.047	−8.80(− 17.40,0.69)	0.071	− 8.80(− 17.59,0.69)	0.068
Both parents OW	− 14.10(− 39.19,21.34)	0.388	− 14.10(− 39.19,21.34)	0.388	−10.05(− 36.61,27.64)	0.550
**Female (*n*** **= 500)**						
Maternal BMI						
NW	Ref		Ref		Ref	
UW	−0.23(−6.24,6.17)	0.915	0.00(−6.03,6.66)	0.972	0.46(−5.81,7.15)	0.899
OW	−5.16(−15.67,6.66)	0.366	−6.67(− 17.21,5.44)	0.267	− 6.46(− 17.01,5.68)	0.281
Paternal BMI						
HW	Ref		Ref		Ref	
OW	−0.46(−9.43,9.65)	0.932	−0.23(− 9.43,9.90)	0.965	0.23(− 9.01,10.15)	0.972
Parents’ weight status combination						
Both parents HW	Ref		Ref		Ref	
OW father, HW mother	−1.14(−10.67,9.14)	0.804	−1.37(− 10.67,9.14)	0.803	−0.92(− 10.26,9.65)	0.867
OW mother, HW father	−6.67(− 17.59,5.68)	0.280	−8.38(−19.46,4.23)	0.179	−8.38(−19.46,4.23)	0.187
Both parents OW	3.99(−23.62,41.91)	0.802	4.71(−23.26,43.22)	0.767	4.95(−23.26,43.55)	0.759

Abbreviation: *BMI* Body mass index, *TL* Telomere length, *UW* Underweight, *NW* Normal weight, *HW* Normal and underweight, *OW* Overweight

Models’ adjustments were according to Table 2

**Table 4:** Associations between parental pre-pregnancy BMI and newborn TL, stratified by paternal age at delivery

Subgroup	Paternal age at delivery (years)			
	< 31(*n* = 496)		≥ 31(*n* = 586)	
	Percentage change (95% CI)	*P*-value	Percentage change (95% CI)	*P*-value
Maternal pre-pregnancy BMI				
NW	Ref		Ref	
UW	3.04(−3.17,9.40)	0.341	−3.62(− 10.05,3.28)	0.292
OW	−18.53(−33.17, − 0.92)	0.041	− 6.46(−13.70,1.62)	0.119
Paternal pre-pregnancy BMI				
HW	Ref		Ref	
OW	−4.72(− 13.90,5.20)	0.337	6.66(−2.95,17.22)	0.184
Parents’ weight status combination*				
Both parents HW	Ref		Ref	
OW father, HW mother	−3.84(−13.30,6.66)	0.451	5.44(−4.50,16.41)	0.302
OW mother, HW father	−19.83(−36.61,1.39)	0.064	−6.24(−13.90,1.86)	0.129

Abbreviation: BMI, body mass index; TL, telomere length; UW, underweight; NW, normal weight; HW, normal and underweight; OW, overweight

Models’ adjustments were according to model B in Table 2

*Both parents OW was excluded for a small number 7(0.6%)

**Table 5:** Associations between parental pre-pregnancy BMI and newborn TL, stratified by maternal age at delivery

Subgroup	Maternal age at delivery (years)			
	< 28(*n* = 978)		≥ 28(*n* = 104)	
	Percentage change (95% CI)	*P*-value	Percentage change (95% CI)	*P*-value
Maternal pre-pregnancy BMI				
NW	Ref		Ref	
UW	−1.37(− 7.10,4.71)	0.647	1.16(− 5.59,8.39)	0.725
OW	−3.17(− 20.93,18.58)	0.756	− 8.38(− 15.67, − 0.46)	0.038
Paternal pre-pregnancy BMI				
HW	Ref		Ref	
OW	−1.37(− 11.08,9.40)	0.798	3.75(− 5.38,13.76)	0.434
Parents’ weight status combination*				
Both parents HW	Ref		Ref	
OW father, HW mother	−0.23(− 10.46,11.17)	0.966	2.09(− 7.10,12.20)	0.675
OW mother, HW father	2.33(−21.84,33.97)	0.866	−9.22(−16.63, − 1.37)	0.023

Abbreviation: *BMI* Body mass index, *TL* Telomere length, *UW* Underweight, *NW* Normal weight, *HW* Normal and underweight, *OW* Overweight

Models’ adjustments were according to model B in Table 2

*Both parents OW was excluded for a small number 7(0.6%)

### Sensitivity analyses

Sensitivity analyses indicated that the results were essentially unchanged after excluding data of mothers who drank before pregnancy, exposed to passive smoking during pregnancy, had pregnancy comorbidities or complications, gave birth to premature or low birth weight newborns, and participants from urban areas (Table 6).

**Table 6:** Sensitivity analyses

	N	Percentage change (95% CI)	*P*-value
**Maternal BMI**			
Model B			
NW	753	Ref	
UW	255	0.23(−4.28,4.95)	0.929
OW	74	−7.96(− 14.69, − 0.69)	0.032
Excluding drinking before pregnancy			
NW	733	Ref	
UW	248	0.93(− 3.62,5.68)	0.678
OW	74	−7.74(−14.49, − 0.69)	0.034
Excluding passive smoking during pregnancy			
NW	294	Ref	
UW	97	−3.84(− 10.67,3.28)	0.285
OW	30	−14.30(− 23.62, − 3.62)	0.010
Excluding pregnancy comorbidities or complications			
NW	659	Ref	
UW	231	0.00(−4.72,4.71)	0.966
OW	60	−10.05(−17.21, −2.28)	0.013
Excluding preterm birth and low birth weight			
NW	709	Ref	
UW	231	0.46(−4.06,5.20)	0.836
OW	72	−7.96(−14.69, −0.69)	0.033
Excluding women from urban areas			
NW	704	Ref	
UW	241	0.69(−3.84,5.44)	0.793
OW	68	−10.05(−16.82, −2.95)	0.007
**Paternal BMI**			
Model B			
HW	995	Ref	
OW	87	0.93(−5.59,8.14)	0.772
Excluding drinking before pregnancy			
HW	972	Ref	
OW	83	−0.23(−6.89,6.91)	0.960
Excluding passive smoking during pregnancy			
HW	396	Ref	
OW	25	−4.50(−15.67,8.14)	0.474
Excluding pregnancy comorbidities or complications			
HW	875	Ref	
OW	75	0.93(−6.24,8.64)	0.793
Excluding preterm birth and low birth weight			
HW	930	Ref	
OW	82	1.39(−5.38,8.89)	0.683
Excluding women from urban areas			
HW	932	Ref	
OW	81	2.57(−4.28,9.90)	0.479
Parents’ weight status combination*			
Model B			
Both parents HW	928	Ref	
OW father, HW mother	80	0.69(−6.24,8.14)	0.866
OW mother, HW father	67	−8.38(−15.47, −0.92)	0.028
Excluding drinking before pregnancy			
Both parents HW	905	Ref	
OW father, HW mother	76	−0.69(−7.53,6.91)	0.860
OW mother, HW father	67	−8.59(−15.47, −1.14)	0.026
Excluding passive smoking during pregnancy			
Both parents HW	368	Ref	
OW father, HW mother	23	−3.62(−15.28,9.65)	0.582
OW mother, HW father	28	−13.30(−23.09, −2.28)	0.021
Excluding pregnancy comorbidities or complications			
Both parents HW	820	Ref	
OW father, HW mother	70	1.16(−6.24,9.14)	0.784
OW mother, HW father	55	−10.05(−17.40, − 1.83)	0.018
Excluding preterm birth and low birth weight			
Both parents HW	865	Ref	
OW father, HW mother	75	1.16(−6.03,8.64)	0.774
OW mother, HW father	65	−8.59(−15.67, −0.92)	0.027
Excluding women from urban areas			
Both parents HW	871	Ref	
OW father, HW mother	74	2.09(−4.94,9.65)	0.575
OW mother, HW father	61	−10.87(−17.78, −3.39)	0.005

Abbreviation: *BMI* Body mass index, *TL* Telomere length, *UW* Underweight, *NW* Normal weight, *HW* Healthy weight (normal and underweight), *OW* Overweight

Models’ adjustments were according to model B in Table 2

Preterm birth was defined as a newborn with gestational age < 37 weeks, low birth weight was defined as a newborn with birth weight < 2500 g

*Both parents OW was excluded for a small number 7(0.6%)

## Discussion

In the present study, we investigated the association of parental pre-pregnancy BMI with newborn TL by using data from the GZBC. We found that mothers who were overweight before pregnancy had significantly shorter cord blood telomere length in their newborns than those who were of normal weight before pregnancy. Further analysis of the combined effects of parental weight status on newborn TL showed that TL was significantly shortened among newborns whose mothers were overweight and fathers were of healthy weight when compared with those whose mothers and fathers were both of normal weight. Subgroup analysis indicated that these effects were more pronounced among male newborns and those whose paternal age < 31 years or maternal age ≥ 28 years.

Overweight and obesity leads to a higher ratio of systemic inflammation and reactive oxygen species (ROS) in adults [25, 26]. Increasing levels of oxidative stress will lead to breakage of DNA and accelerated shortening of telomeres [27, 28]. Parental obesity has been linked to a range of adverse effects on newborn health outcomes. Higher maternal BMI is accompanied with a higher inflammatory and oxidative stress intrauterine environment for the developing fetus [29]. The changed intrauterine environment will lead to an adverse impact on telomere biology during the utero life and affect fetal programming [30]. The results of mothers in our study were similar to a recent study conducted by Dries S et al. [17]. However, they found cord blood TL were not significantly lower in overweight mothers compared to normal weight mothers, even in the unadjusted model. That might be explained by ethnicity-specific differences. Asians have a higher percentage of body fat compared to Caucasians with the same BMI [31].

We did not observe any significant association between paternal pre-pregnancy BMI and newborn TL, though most studies had concluded that male obesity had an inverse association with the quality of sperm [32–34] and the health outcomes of the next generation [35, 36]. The effect of males being overweight does not appear to be great [35, 37, 38], and it implied that the effect of maternal pre-pregnancy BMI on newborn TL was dominant. The phenomenon of parental BMI on newborn TL might be explained by the greater influence of mothers through the whole course of pregnancy. On the other hand, some studies reported that paternal effects on the next generation emerged later [4, 36]. There were no obese fathers in our current study, therefore, it was understandable to have such a result between paternal BMI and newborn TL.

Stratified analysis showed that the effect of higher maternal pre-pregnancy BMI on male newborn TL was stronger than that of female newborns, Reyes et al. reported that the level of estrogen [39] among female fetuses is higher than male fetuses. Higher level of estrogen leads to higher antioxidant enzymes level by up-regulating superoxide dismutase and glutathione peroxidase gene expression [40]. Therefore, male fetuses might be more susceptible to oxidative stress caused by higher maternal pre-pregnancy BMI. The stratified analysis also indicated that the inverse association between maternal overweight and newborn TL was more pronounced among mothers ≥28 years or fathers < 31 years. Previous studies reported that newborn TL tended to be longer with increased paternal age [6]. Our current results implied that the positive effects of increased paternal age at delivery and the negative effects of maternal higher BMI before pregnancy on newborn TL might be additive. It was reported that there was an increased risk of adverse perinatal outcomes with higher maternal age at delivery [41, 42]. Therefore, it was understandable that higher maternal age at delivery might increase the effect of maternal pre-pregnancy overweight on newborn TL. Besides, TL is shortened during each cell division, and it means that older fathers tend to have shorter TL generally. Longitudinal studies showed that an annual loss between 32.2 and 45.5 bp is estimated in adult leukocytes [43], but, Wright et al. ever reported that germ line cells didn’t show telomere shortening [44]. Therefore, the effect of parental age at delivery on newborn TL is still a controversial topic and further research is needed to determine the relationship between newborn TL and parental age at delivery. In the sensitivity analyses, our associations remained unchanged after adjusting for different covariates and potential cofounders and persisted after excluding major confounders, further suggesting an independent association between maternal overweight and newborn TL.

To our knowledge, our study was the first to investigate the association between parental pre-pregnancy BMI and newborn TL in Asia and was based on a relatively large sample size. The cohort is composed of citizens originally from Guangxi province and there was an advantage to analyzing homogeneous populations. In addition, the participants from 6 different counties gave our study a certain generalization for other populations. The very low number of smoking women was also a strength of our study, which enabled us to analyze the data without this confounding effects. However, our research had several limitations should be noted. First, parental weight and height were self-reported, which might lead to recall bias. Second, we did not adjust for some confounding factors, such as parental education, maternal prenatal folate concentration and enough paternal environmental lifestyle factors before pregnancy. These factors were reported to be associated with newborn TL or birth outcomes [45–48]. Third, although BMI is considered as a good measure of assessing overweight and obesity in adults, waist circumference, hip circumference, total body fat, and visceral adipose tissue volume [49] are also recommended to assess overweight and obesity. Fourth, the number of overweight participants in our study was relatively small, however, the socioeconomic status of Guangxi province [18] and the evidence from previous study [50] showed that the proportion of overweight participants in our study was understandable. Lastly, previous studies showed that leukocyte TL was inversely associated with BMI in adulthood [51], and there was a strong correlation between newborn TL in cord blood and both parental TL [17]. Therefore, the association between pre-pregnancy maternal BMI and newborn TL might be mediated by parental TL. Further precise research is needed to explore the relationship between parental pre-pregnancy BMI and newborn TL.

## Conclusion

In conclusion, maternal overweight, not paternal overweight before pregnancy may shorten newborn telomere length. The trend of rapid economic growth and urbanization has a great influence on the Chinese population, and this includes the adoption of a sedentary lifestyle and the high availability of foods with high caloric content. Hence, our results comprise of an important public health finding. Weight control in reproductive women and effective healthy weight management before pregnancy may be of particular benefit for improving longevity and life quality of offspring.

## Supplementary Information

**Additional file 1: Figure S1.** Flowchart of the study parent-newborn pairs.

**Additional file 2: Figure S2.** Pearson correlation between parental pre-pregnancy BMI and newborn telomere length. (Relative average telomere lengths were expressed as the ratio of telomere copy number to single-copy gene number (T/S ratio). a Maternal BMI. b Paternal BMI).

**Additional file 3: Figure S3.** Restricted cubic spline analysis of the correlation between parental BMI and newborn telomere length. (Relative average telomere lengths were expressed as the ratio of telomere copy number to single-copy gene number (T/S ratio). c. Maternal BMI (knots = 3). d. Paternal BMI (knots = 6).

**Additional file 4: Table S1.** Categorized analysis between parental pre-pregnancy BMI and newborn TL (*n* = 645).

**Additional file 5: Table S2.** Interaction between parental pre-pregnancy BMI and newborn sex or parental age at delivery.

## Data Availability

The datasets for the current study are available from the corresponding author on reasonable request.

## Abbreviations

TL: Telomere length
BMI: Body mass index
T/S: Telomere copy number/single-copy gene number
qPCR: Quantitative real-time polymerase chain reaction
CI: Confidence interval
SD: Standard deviation
IQR: Interquartile range
ROS: Reactive oxygen species
UW: Underweight
NW: Normal weight
OW: Overweight
HW: Healthy weight (normal and underweight)
AIC: Akaike information criterion
