# Supplementary material for: Maternal overweight but not paternal overweight before pregnancy is associated with shorter newborn telomere length: evidence from Guangxi Zhuang birth cohort in China
Source: BMC Pregnancy Childbirth. 2021 Apr 9;21:283. doi: 10.1186/s12884-021-03757-x (PMC8033662; doi:10.1186/s12884-021-03757-x)
Supplement: Supplementary file 1 — Additional file 1: Figure S1. Flowchart of the study parent-newborn pairs. [file 12884_2021_3757_MOESM1_ESM.docx]

Parent-newborn pairs recruited from June, 2015 to Dec, 2016(n=1239)

- Excluded twins (n=15) and stillbirth (n=9)

Included parent-newborn pairs(n=1215)

- No availability of quality cord blood DNA (n=69)
- No availability of cord blood telomere measurements (n=26)
- No availability of data on parental height or weight (n=38)

Parent-newborn pairs available with full data and cord blood telomeres(n=1082)

**Figure S1.** Flowchart of the study parent-newborn pairs
