# Supplementary material for: Maternal overweight but not paternal overweight before pregnancy is associated with shorter newborn telomere length: evidence from Guangxi Zhuang birth cohort in China
Source: BMC Pregnancy Childbirth. 2021 Apr 9;21:283. doi: 10.1186/s12884-021-03757-x (PMC8033662; doi:10.1186/s12884-021-03757-x)
Supplement: Supplementary file 2 — Additional file 2: Figure S2. Pearson correlation between parental pre-pregnancy BMI and newborn telomere length. (Relative average telomere lengths were expressed as the ratio of telomere copy number to single-copy gene number (T/S ratio). a Maternal BMI. b Paternal BMI). [file 12884_2021_3757_MOESM2_ESM.docx]

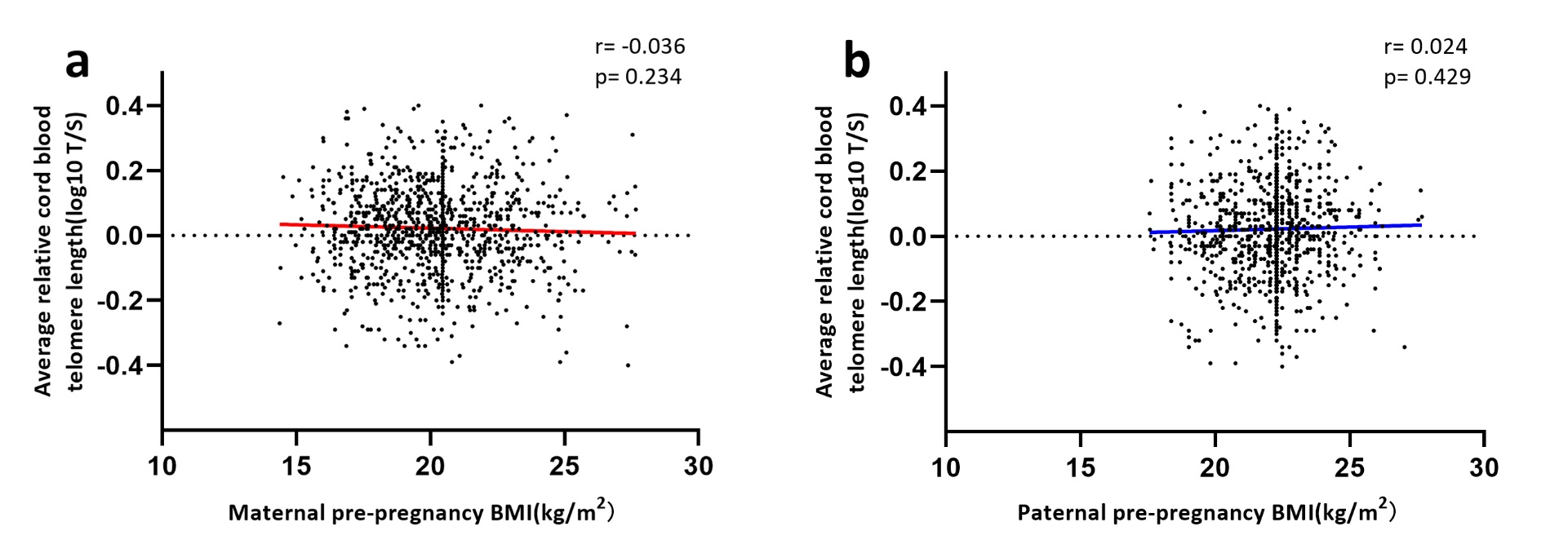


**Figure S2.** Pearson correlation between parental pre-pregnancy BMI and newborn telomere length. (Relative average telomere lengths were expressed as the ratio of telomere copy number to single-copy gene number (T/S ratio). a Maternal BMI. b Paternal BMI).
