# Supplementary material for: Maternal overweight but not paternal overweight before pregnancy is associated with shorter newborn telomere length: evidence from Guangxi Zhuang birth cohort in China
Source: BMC Pregnancy Childbirth. 2021 Apr 9;21:283. doi: 10.1186/s12884-021-03757-x (PMC8033662; doi:10.1186/s12884-021-03757-x)
Supplement: Supplementary file 3 — Additional file 3: Figure S3. Restricted cubic spline analysis of the correlation between parental BMI and newborn telomere length. (Relative average telomere lengths were expressed as the ratio of telomere copy number to single-copy gene number (T/S ratio). c. Maternal BMI (knots = 3). d. Paternal BMI (knots = 6). [file 12884_2021_3757_MOESM3_ESM.docx]

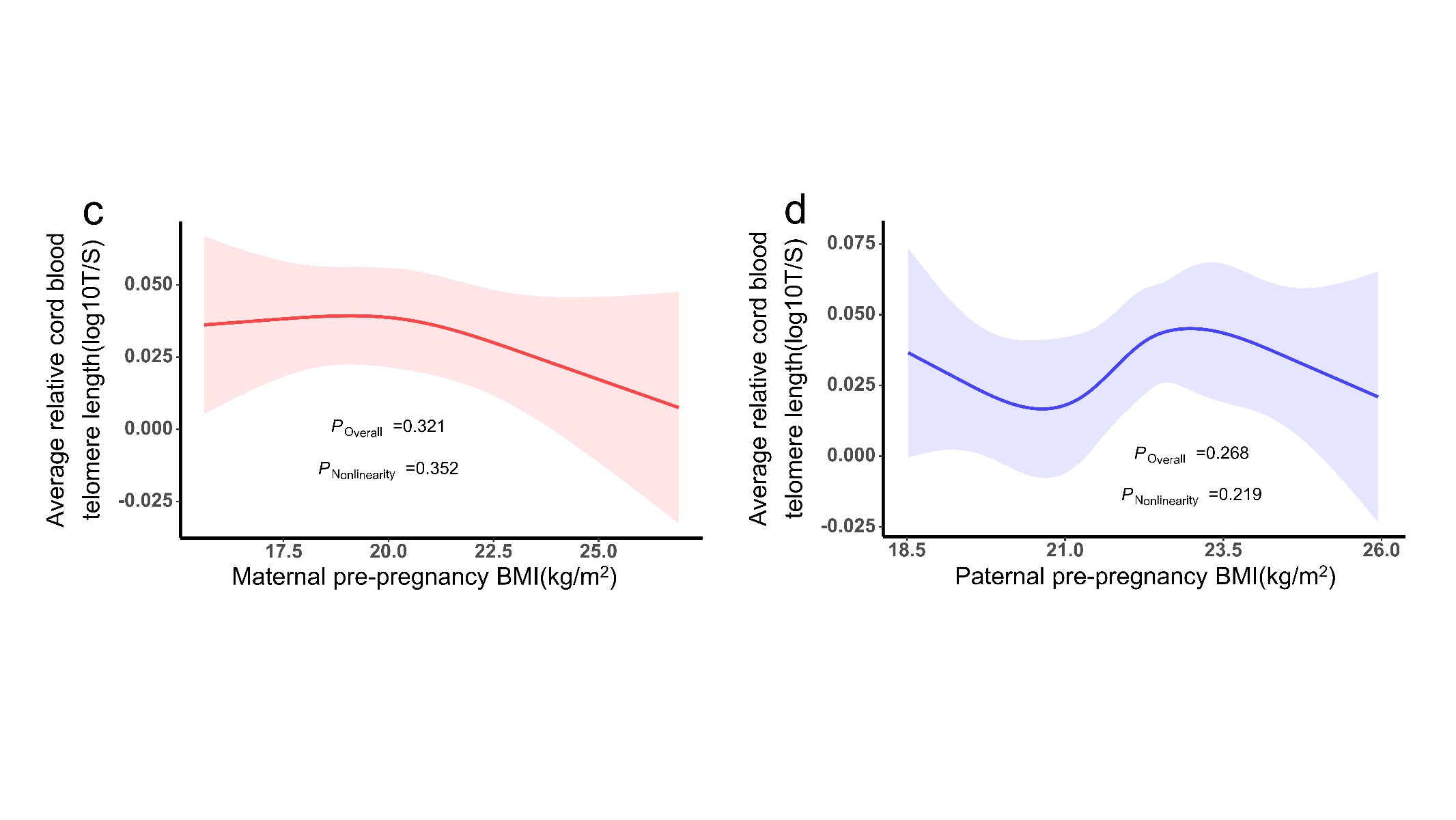


**Figure S3.** Restricted cubic spline analysis of the correlation between parental BMI and newborn telomere length. (Relative average telomere lengths were expressed as the ratio of telomere copy number to single-copy gene number (T/S ratio). c. Maternal BMI (knots=3). d. Paternal BMI (knots=6).

The number of knots was chosen based on the Akaike information criterion (AIC).

Models adjusted for parental age, newborn factors (sex, gestational age and birth weight). maternal factors (residential place, gravidity, parity, drinking before pregnancy, passive smoking during pregnancy, pregnancy comorbidities or complications, and cesarean section).
