# Supplementary material for: Maternal overweight but not paternal overweight before pregnancy is associated with shorter newborn telomere length: evidence from Guangxi Zhuang birth cohort in China
Source: BMC Pregnancy Childbirth. 2021 Apr 9;21:283. doi: 10.1186/s12884-021-03757-x (PMC8033662; doi:10.1186/s12884-021-03757-x)
Supplement: Supplementary file 4 — Additional file 4: Table S1. Categorized analysis between parental pre-pregnancy BMI and newborn TL (n = 645). [file 12884_2021_3757_MOESM4_ESM.docx]

**Table S1** Categorized analysis between parental pre-pregnancy BMI and newborn TL

|  | Unadjusted | | Model A | | Model B | |
| --- | --- | --- | --- | --- | --- | --- |
| All (n =645) | Percentage change (95% CI) | *P*-value | Percentage change (95% CI) | *P*-value | Percentage change (95% CI) | *P*-value |
| Maternal BMI |  | |  | |  | |
| NW (n =437) | Ref | | Ref | | Ref | |
| UW (n =163) | -0.92(-6.24,4.71) | 0.739 | -0.01(-5.59,5.93) | 0.996 | -0.02(-5.59,5.93) | 0.994 |
| OW (n =45) | -7.74(-16.05,1.16) | 0.089 | -7.74(-16.25,1.62) | 0.101 | -7.53(-16.05,1.86) | 0.117 |
| Paternal BMI |  |  |  |  |  |  |
| HW (n =577) | Ref |  | Ref |  | Ref |  |
| OW (n =68) | 2.57(-5.16,10.66) | 0.523 | 2.57(-5.16,10.92) | 0.527 | 2.57(-5.16,10.92) | 0.524 |
| Parents’ weight status combination | | | | | | |
| Both parents HW (n =537) | Ref |  | Ref |  | Ref |  |
| OW father, HW mother (n =63) | 2.57(-5.38,11.17) | 0.545 | 2.57(-5.38,11.17) | 0.546 | 2.57(-5.59,11.17) | 0.553 |
| OW mother, HW father (n =40) | -7.74(-16.44,1.86) | 0.110 | -7.74(-16.63,2.09) | 0.123 | -7.53(-16.44,2.57) | 0.135 |
| Both parents OW (n =5) | -4.5(-27.05,25.31) | 0.742 | -5.59(-28.06,23.88) | 0.675 | -4.72(-27.39,25.31) | 0.732 |

Abbreviation: BMI, body mass index; TL, telomere length; UW, underweight; NW, normal weight; HW, healthy weight (normal and underweight); OW, overweight.

Model A: adjusted for parental age, newborn factors (sex, gestational age and birth weight).

Model B: Model A + maternal factors (residential place, gravidity, parity, drinking before pregnancy, passive smoking during pregnancy, pregnancy comorbidities or complications, and cesarean section) + paternal factors (drinking before pregnancy, smoking before pregnancy).

Estimates are presented as a percentage change in average relative telomere length.
