# Supplementary material for: Maternal overweight but not paternal overweight before pregnancy is associated with shorter newborn telomere length: evidence from Guangxi Zhuang birth cohort in China
Source: BMC Pregnancy Childbirth. 2021 Apr 9;21:283. doi: 10.1186/s12884-021-03757-x (PMC8033662; doi:10.1186/s12884-021-03757-x)
Supplement: Supplementary file 5 — Additional file 5: Table S2. Interaction between parental pre-pregnancy BMI and newborn sex or parental age at delivery. [file 12884_2021_3757_MOESM5_ESM.docx]

**Table** **S2** Interaction between parental pre-pregnancy BMI and newborn sex or parental age at delivery

| Categorical variables | *P*-value for interaction | | |
| --- | --- | --- | --- |
|  | Newborn sex | Paternal age at delivery  (31 years) | Maternal age at delivery  (28 years) |
| Maternal pre-pregnancy BMI | 0.712 | 0.989 | 0.532 |
| Paternal pre-pregnancy BMI | 0.713 | 0.106 | 0.417 |
| Parents’ weight status combination | 0.523 | 0.242 | 0.127 |

Abbreviation: BMI, body mass index.

Models’ adjustments were according to model B in Table 2.
